# Clinical Insights Into Bloom Syndrome With Dentinogenesis Imperfecta: A Report of a Rare Case

**DOI:** 10.7759/cureus.116280

**Published:** 2026-09-15

**Authors:** Sindhuja Rajalingam, Ramachandra Reddy Gowda Venkatesha, Karthik Rajaram Mohan, Saramma Mathew Fenn, Reethika Rathan

**Affiliations:** 1 Oral Medicine and Radiology, Vinayaka Mission's Sankarachariyar Dental College, Vinayaka Mission's Research Foundation, Salem, IND

**Keywords:** bloom-syndrome, flat head, high arched palate, hypoplastic uvula, plagiocephaly, telengiectasia

## Abstract

Bloom syndrome, a rare autosomal recessive genetic disorder, is characterized by a predisposition to various types of cancer, a distinctive facial appearance, and a range of other physical and developmental abnormalities. Individuals afflicted with this condition typically exhibit proportional growth deficiency, with adult heights rarely exceeding 5 feet and characteristic narrow facies with a prominent nose and ears. The syndrome is also marked by photosensitive facial skin, which can be exacerbated by exposure to sunlight, and an increased susceptibility to respiratory tract infections and other bacterial infections due to an underlying immune deficiency. The clinical presentation of Bloom syndrome is multifaceted and often includes a high-pitched voice, hypogonadism, and infertility, particularly in male patients. Additionally, patients may develop chronic lung disease and adult-onset diabetes mellitus as they age. The clinical diagnosis of Bloom syndrome is typically confirmed through cytogenetic analysis, which reveals a characteristic chromosome instability and an increased rate of somatic mutations. This case report provides a comprehensive understanding of the various aspects of this rare syndrome, offering a reliable and thorough resource for medical professionals, researchers, and students interested in genetic disorders. The hallmark clinical features, including growth retardation, facial characteristics, and immune deficiency, have been well-documented in several case reports and studies. The increased cancer risk, a defining feature of Bloom syndrome, is also extensively discussed, with patients being susceptible to a wide range of cancers, often at an unusually early age. While ocular manifestations are rarely reported, a case of Bloom syndrome with dentinogenesis imperfecta has been described, highlighting the potential for diverse clinical presentations within this disorder.

## Introduction

Bloom syndrome, a rare genetic disorder, is characterized by distinctive physical and medical features, including increased susceptibility to a wide range of cancers, short stature, photosensitivity, and a characteristic facial appearance [1]. This complex condition, first identified in 1954, has continued to captivate the attention of researchers and clinicians alike as they strive to unravel its underlying mechanisms and develop effective management strategies. One of the hallmarks of Bloom syndrome is the heightened risk of developing various types of cancer, including leukemia, lymphoma, and solid tumors [1,2]. This increased vulnerability is attributed to a defective DNA repair mechanism, which leads to genetic instability and the accumulation of mutations. The precise molecular pathways involved in this process have been the subject of extensive investigation, with studies revealing the crucial role of the Bloom helicase, a protein essential for maintaining genomic integrity [1]. The clinical manifestations of Bloom syndrome extend beyond the heightened cancer risk, with affected individuals often experiencing a range of other health complications. These may include growth retardation, immunodeficiency, and metabolic abnormalities, reflecting the widespread impact of the genetic defect on multiple physiological systems. The genetic basis of Bloom syndrome has been elucidated, with mutations in the helicase (BLM) gene on chromosome 15 identified as the primary cause of the disorder.

## Case presentation

A 14-year-old boy reported to our department for a routine dental checkup. A general examination revealed his vitals are stable. His family history revealed that he was born to parents of a consanguineous marriage. He gave no history of forceps delivery or vacuum extractor during his birth and no history of harmful oral habits, such as chewing or smoking tobacco. Extraoral examination revealed a long narrow dolichocephalic skull, multiple discrete hyperpigmentations on the facial skin, a narrow nose, flat head occiput, and plagiocephaly (Figures 1A-1B).

**Figure 1:**
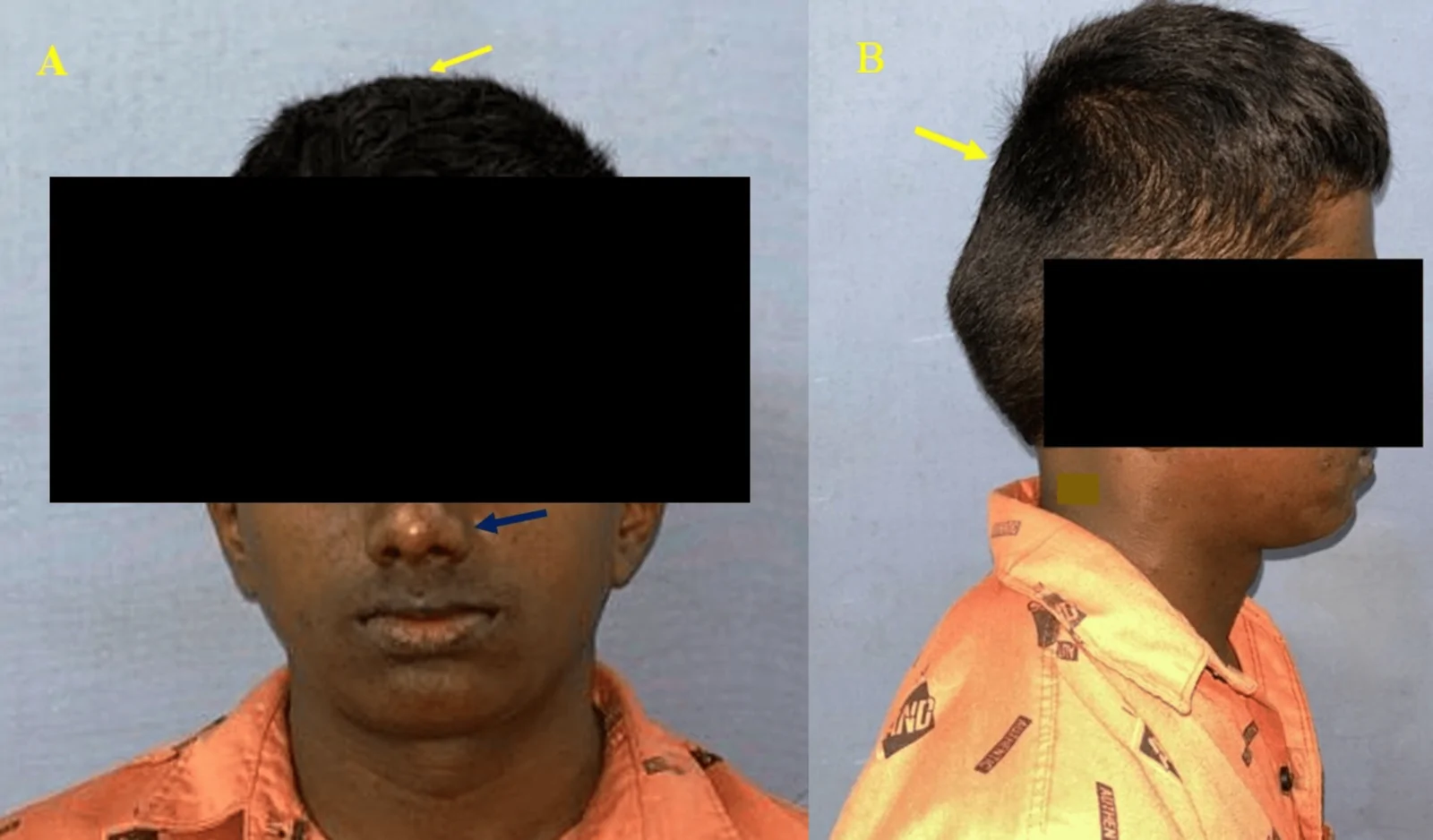
(A) Frontal profile revealed a flat head (yellow arrow) and narrow face and nose (blue arrow); (B) lateral profile view revealed a flat head plagiocephaly (yellow arrow).

His flexor aspects of the palms revealed multiple discrete hyperpigmentation (Figure 2).

**Figure 2:**
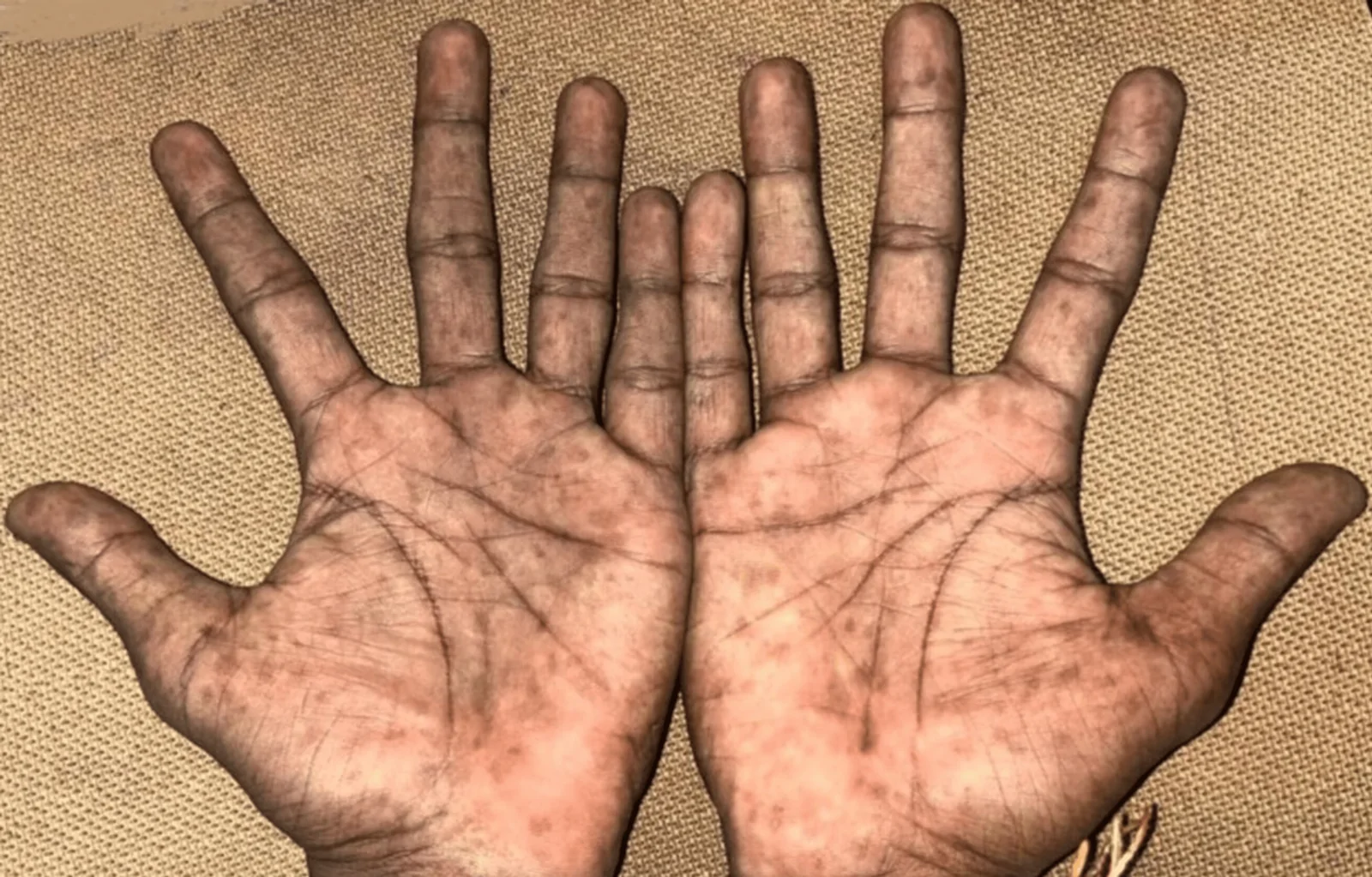
Examination of flexor aspects of hands revealed multiple discrete pigmentations.

The hard tissue examination revealed a generalized mild grayish opalescent hue on all his teeth (Figure 3).

**Figure 3:**
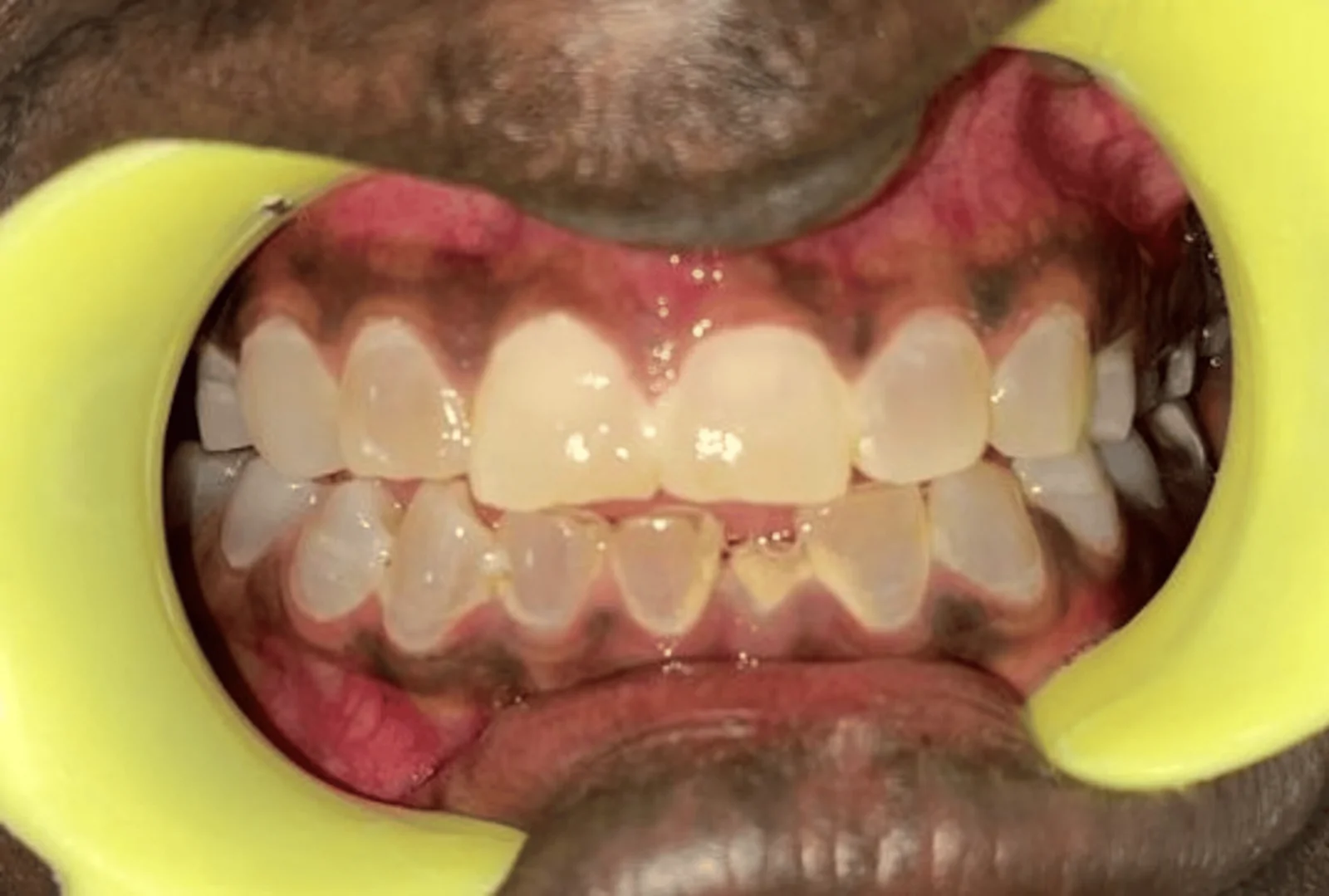
Intraoral hard tissue examination revealed a grayish hue on all the teeth,

Orthopantomography revealed a lack of differentiation in the radiodensities of enamel and dentin, as their densities remained the same. Shell-like teeth, due to a thin layer of dentin, resulted in enlarged pulp chambers, suggestive of dentinogenesis imperfecta. Additional findings included generalized alveolar bone loss due to periodontitis, impacted maxillary and mandibular third molars, and dental caries with pulp involvement in the right maxillary second premolar and first molar (Figure 4).

**Figure 4:**
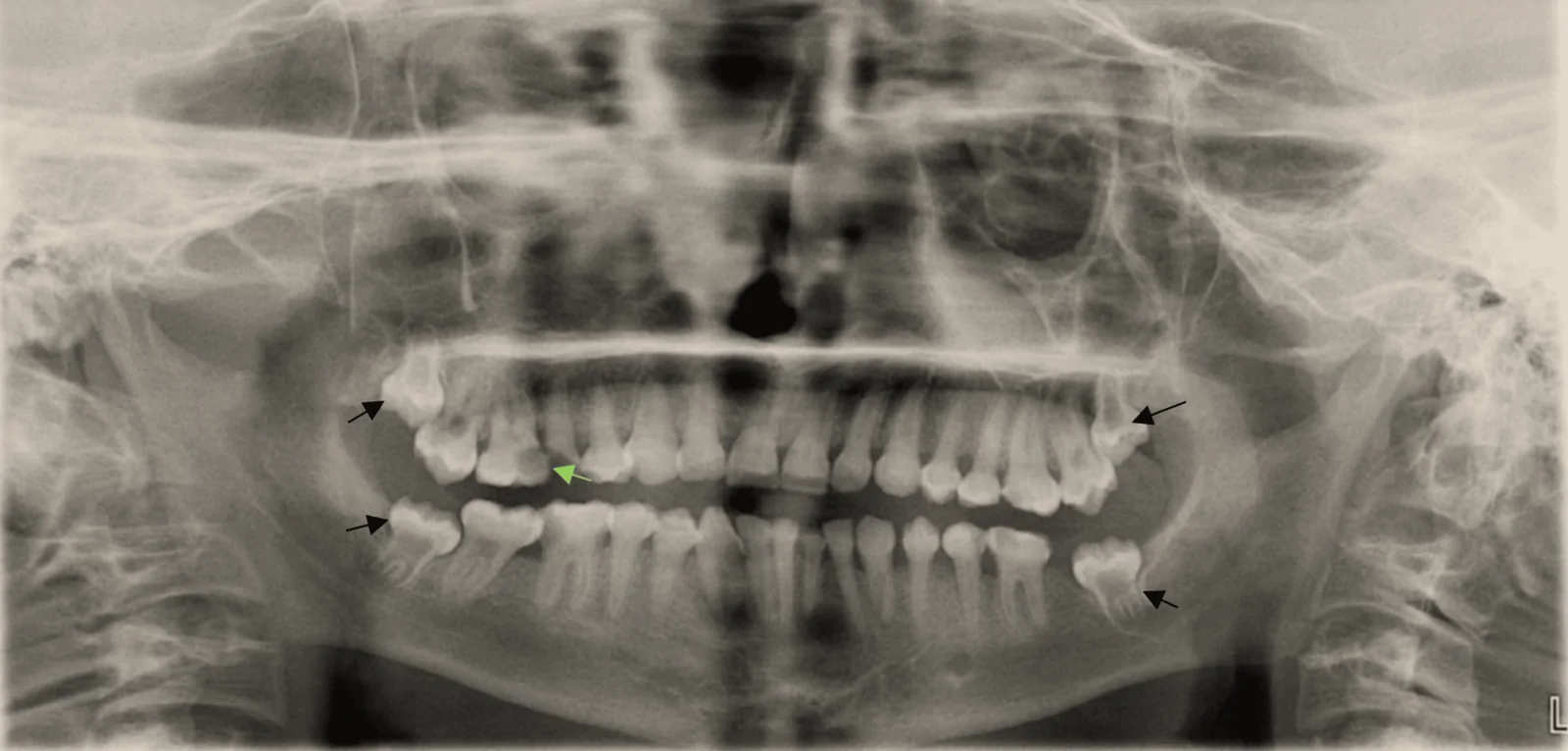
Orthopantomography revealed a lack of differentiation in the radiodensities of enamel and dentin, as their densities remained the same. Shell-like teeth were observed due to a thin layer of dentin, resulting in enlarged pulp chambers suggestive of dentinogenesis imperfecta. Impacted third molars (black arrows) and dental caries with pulp involvement in the right maxillary second premolar and first molar (green arrow) are also noted.

Intraoral soft tissue examination revealed macroglossia and hypoplastic uvula (Figures 5A-5B).

**Figure 5:**
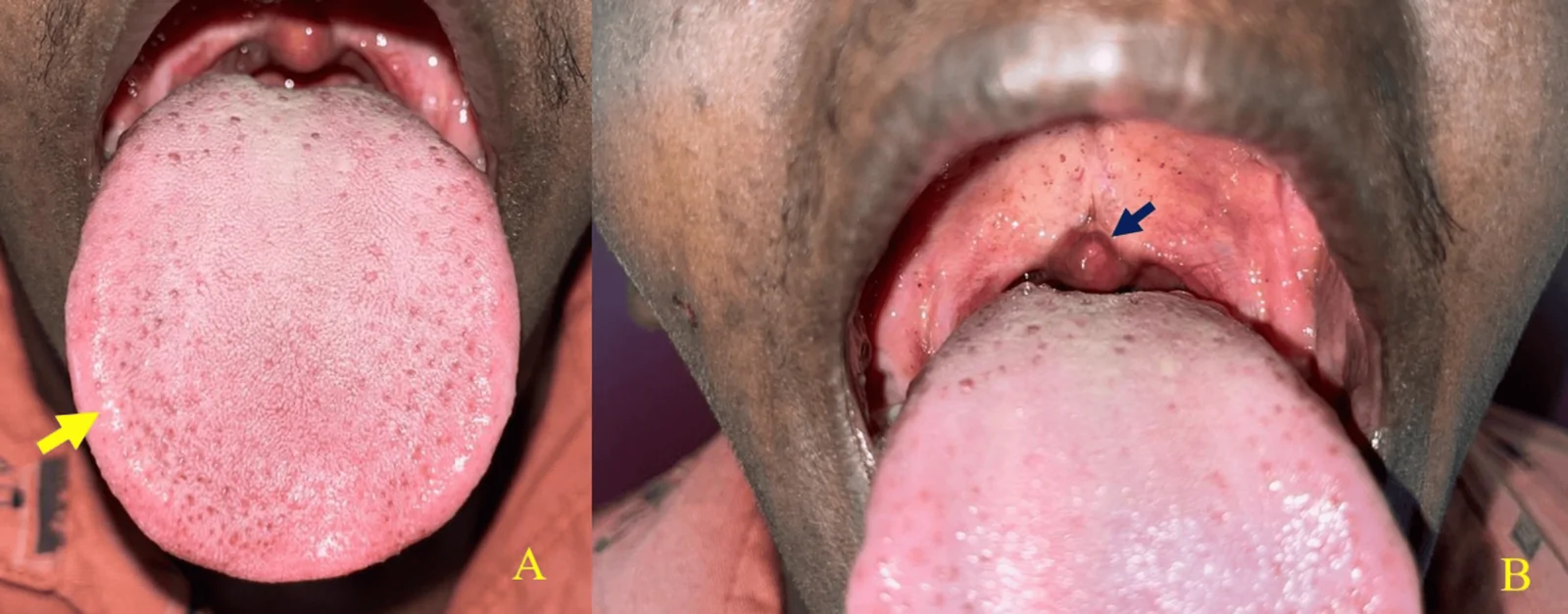
(A) Intraoral examination revealed macroglossia (yellow arrow) and (B) hypoplastic uvula (blue arrow).

The differential diagnosis for dentinogenesis imperfecta includes amelogenesis imperfecta, in which open contact points occur in the interdental areas of the teeth, and enamel appears malformed. In dentinogenesis imperfecta, no such malformations occur in the surface enamel. The differential diagnosis for macroglossia includes Down syndrome. Patients with Down syndrome have short stubby fingers with increased simian crease and low intelligence quotient (IQ). No such alterations exist in our case. Based on the above clinical and radiographic findings, a final diagnosis of Bloom syndrome associated with dentinogenesis imperfecta was made.

## Discussion

Bloom syndrome is an autosomal recessive genetic disorder characterized by short stature, photosensitivity to sun exposure, genomic instability, and a predisposition to the development of a variety of cancers, such as non-Hodgkin's and Hodgkin's lymphomas, carcinomas of the respiratory tract, non-small cell lung carcinoma, colorectal carcinoma, liver carcinoma, breast carcinoma, squamous cell and basal cell carcinoma of the skin, osteosarcoma, retinoblastoma, and brain tumors [1,2]. One of the hallmarks of Bloom syndrome is the heightened risk of developing various types of cancer, including leukemia, lymphoma, and solid tumors [1]. This increased vulnerability is attributed to a defective DNA repair mechanism, which leads to genetic instability and the accumulation of mutations. The precise molecular pathways involved in this process have been the subject of extensive investigation, with studies revealing the crucial role of the Bloom helicase, a protein essential for maintaining genomic integrity [1]. The clinical manifestations of Bloom syndrome extend beyond the heightened cancer risk, with affected individuals often experiencing a range of other health complications. These may include growth retardation, immunodeficiency, and metabolic abnormalities, reflecting the widespread impact of the genetic defect on multiple physiological systems. The genetic basis of Bloom syndrome has been elucidated, with mutations in the Bloom syndrome helicase (BLM) gene, located on chromosome 15, being the primary cause of the disorder.

Bloom syndrome shares certain phenotypic similarities with other chromosomal abnormalities, such as trisomy 21 [2]. This observation has led researchers to explore potential shared pathways and mechanisms that may underlie these related genetic conditions, offering valuable insights into the complex interplay between genotype and phenotype. Managing Bloom syndrome requires a multidisciplinary approach involving close monitoring, early detection of cancer, and tailored interventions to address the diverse range of symptoms. The development of targeted therapies, such as those aimed at modulating the DNA repair machinery or regulating the Bloom helicase, holds promise for improving the quality of life and prognosis for individuals with this challenging disorder. Ongoing research into the molecular mechanisms underlying Bloom syndrome continues to shed light on the broader implications of this condition, not only for the affected individuals but also for our understanding of genome stability and the complex interplay between genetic and environmental factors in disease development.

Orofacial manifestations of Bloom syndrome

Bloom syndrome, a rare autosomal recessive disorder, is characterized by a distinctive facial appearance, photosensitivity of the skin, and an increased risk of various types of cancer. While the genetic and systemic aspects of this condition have been extensively studied, the orofacial manifestations of Bloom syndrome warrant closer examination. Bloom syndrome is associated with conjunctival telangiectasias [3]. Additionally, the photosensitive skin lesions on the face can extend to the oral mucosa, leading to erythematous and telangiectatic changes within the oral cavity [4]. One of the hallmark features of Bloom syndrome is the characteristic facial appearance, which includes a long, narrow face, prominent nose, and micrognathia. These craniofacial abnormalities can lead to various orofacial complications, such as high-arched palate, malocclusion, and temporomandibular joint dysfunction [5]. Patients with Bloom syndrome are also at an increased risk of developing various types of cancer, including those of the head and neck region. These cancers can manifest as oral lesions, such as leukoplakia, erythroplakia, or even oropharyngeal carcinoma. Furthermore, the immunodeficiency associated with Bloom syndrome can predispose these patients to recurrent bacterial infections, affecting the oral cavity and leading to diseases like chronic periodontitis or osteomyelitis of the jaw bones [5]. Careful monitoring and management of the orofacial manifestations of Bloom syndrome are crucial for maintaining the overall health and well-being of affected individuals. Regular dental checkups, oral hygiene instructions, and prompt treatment of any oral complications can help mitigate the impact of this rare syndrome on the orofacial region [6,7,8]. Refractory duodenal ulcer is a rare complication in Bloom syndrome [9]. Bloom syndrome is associated with growth hormone deficiency and pancreatic carcinoma [10,11]. Acute myeloid leukemia from myelodysplastic syndrome can occur as a complication of Bloom syndrome [12,13].

Clinical significance

The case report describes a rare occurrence of dentinogenesis imperfecta in a patient with Bloom syndrome. Patients with dentinogenesis imperfecta must also be screened for Bloom syndrome. Dentinogenesis imperfecta is an autosomal dominant disorder that affects the development of dentin, the tooth's inner layer. Individuals with this condition often have teeth that are discolored, brittle, and prone to premature wear and loss. The coexistence of Bloom syndrome and dentinogenesis imperfecta in a single patient is infrequent. The exact mechanisms underlying the development of dentinogenesis imperfecta in Bloom syndrome are not fully understood. The chromosomal instability and increased genetic mutations associated with Bloom syndrome may have disrupted the normal processes of tooth development, leading to the observed dental abnormalities. Alternatively, the shared genetic origins of these two conditions may have contributed to their co-occurrence in this patient. Regardless of the underlying mechanisms, identifying this rare association highlights the importance of comprehensive oral health assessments in individuals with Bloom syndrome, as they may be at increased risk for developing dentinogenesis imperfecta and other dental complications.

## Conclusions

Bloom syndrome is a complex and challenging disorder that requires a multidisciplinary approach to management. Careful monitoring and management of the orofacial manifestations of Bloom syndrome are crucial for maintaining the overall health and well-being of affected individuals. Regular dental checkups, oral hygiene instructions, and prompt treatment of any oral complications can help mitigate the impact of this rare syndrome on the orofacial region. Despite the significant medical challenges these patients face, ongoing research and advancements in our understanding of the underlying genetic mechanisms may lead to developing more targeted therapeutic interventions in the future.

## References

[1] Campbell MB, Campbell WC, Rogers J (2018). Bloom syndrome: research and data priorities for the development of precision medicine as identified by some affected families. Cold Spring Harb Mol Case Stud.

[2] Kharat SS, Tripathi V, Damodaran AP (2016). Mitotic phosphorylation of Bloom helicase at Thr182 is required for its proteasomal degradation and maintenance of chromosomal stability. Oncogene.

[3] MacLean GA, McEldoon J, Huang J, Allred J, Canver MC, Orkin SH (2018). Downregulation of endothelin receptor b contributes to defective b cell lymphopoiesis in trisomy 21 pluripotent stem cells. Sci Rep.

[4] Sahn EE, Hussey RH 3rd, Christmann LM (1997). A case of Bloom syndrome with conjunctival telangiectasia. Pediatr Dermatol.

[5] Wong BJ, Hashisaki GT (1996). Treatment of Bloom syndrome patients: guidelines and report of a case. Otolaryngol Head Neck Surg.

[6] Gould NJ, Holjak EJ, Barat J, Tay KY, Dar AR (2024). Bloom syndrome complicated by low-grade lymphoma and non-small cell lung cancer: a case report. Cureus.

[7] Bhisitkul RB, Rizen M (2004). Bloom syndrome: multiple retinopathies in a chromosome breakage disorder. Br J Ophthalmol.

[8] Zhang J, Wei C, Wu J (2024). Ischemic stroke with positive antiphospholipid antibodies in bloom syndrome: a case report. J Stroke Cerebrovasc Dis.

[9] Usami M, Ikawa Y, Sakai Y, Fujiki T, Wada T (2022). Refractory gastroduodenal ulcers: a rare complication with Bloom syndrome. Clin Case Rep.

[10] Verpula M, Danda VS, Paidipally SR, Konda C (2020). Bloom's syndrome with growth hormone deficiency: a rare association. BMJ Case Rep.

[11] Tzfoni I, Chayo J, Shaked M, Bernstein E, Dekel R, Arber N, Shapira S (2019). Pancreatic cancer in bloom syndrome. SAGE Open Med Case Rep.

[12] Gürbüz AF, Eryılmaz MK, Yıldız O, Kılınç F, Araz M, Artaç M (2024). Rare case of myelodysplastic syndrome with excess blasts 2 developing after adjuvant chemoradiotherapy for triple-negative breast cancer in a patient with Bloom syndrome. Strahlenther Onkol.

[13] Aljarad S, Alhamid A, Rahmeh AR (2018). Bloom syndrome with myelodysplastic syndrome that was converted into acute myeloid leukaemia, with new ophthalmologic manifestations: the first report from Syria. Oxf Med Case Reports.

